# Managing Conflict between Bats and Humans: The Response of Soprano Pipistrelles (*Pipistrellus pygmaeus*) to Exclusion from Roosts in Houses

**DOI:** 10.1371/journal.pone.0131825

**Published:** 2015-08-05

**Authors:** Emma Stone, Matt R. K. Zeale, Stuart E. Newson, William J. Browne, Stephen Harris, Gareth Jones

**Affiliations:** 1 School of Biological Sciences, Life Sciences Building, University of Bristol, 24 Tyndall Avenue, Bristol, BS8 1TQ, United Kingdom; 2 British Trust for Ornithology, The Nunnery, Thetford, Norfolk, IP24 2PU, United Kingdom; 3 Graduate School of Education, and Centre for Multilevel Modelling, University of Bristol, 2 Priory Road, Bristol, BS8 1TX, United Kingdom; Università degli Studi di Napoli Federico II, ITALY

## Abstract

Conflict can arise when bats roost in human dwellings and householders are affected adversely by their presence. In the United Kingdom, the exclusion of bats from roosts can be licensed under exceptional circumstances to alleviate conflict, but the fate of excluded bats and the impact on their survival and reproduction is not well understood. Using radio-tracking, we investigated the effects of exclusion on the soprano pipistrelle *Pipistrellus pygmaeus*, a species that commonly roosts in buildings in Europe. Exclusions were performed under licence at five roosts in England in spring, when females were in the early stages of pregnancy. Following exclusion, all bats found alternative roosts and colonies congregated in nearby known roosts that had been used by radio-tagged bats prior to exclusion. We found no difference in roosting behaviour before and after exclusion. Both the frequency of roost switching and the type of roosts used by bats remained unchanged. We also found no change in foraging behaviour. Bats foraged in the same areas, travelled similar distances to reach foraging areas and showed similar patterns of habitat selection before and after exclusion. Population modelling suggested that any reduction in survival following exclusion could have a negative impact on population growth, whereas a reduction in productivity would have less effect. While the number of soprano pipistrelle exclusions currently licensed each year is likely to have little effect on local populations, the cumulative impacts of licensing the destruction of large numbers of roosts may be of concern.

## Introduction

Many bat species roost in buildings or other man-made structures [1–5]. In Europe, soprano pipistrelles *Pipistrellus pygmaeus* and common pipistrelles *P*. *pipistrellus* are so well adapted to man-made sites that they are rarely found in natural roosts [2,6,7]. While some species of bats have probably benefitted from the increased roosting opportunities provided by human development [8,9], roosts in buildings are at increased risk of disturbance.

In the United Kingdom, bats are strictly protected under European and national legislation due to concerns over their conservation status. The Conservation of Habitats and Species Regulations 2010 protects all bat roosts from destruction, damage or disturbance, whether occupied or not. Where householders are severely affected by bat roosts in their dwelling, the Department for Environment, Food and Rural Affairs (Defra) authorises Statutory Nature Conservation Organisations (SNCOs) to grant licences for management and mitigation activities to help resolve the conflict. The legislation assumes that licensed activities will not be detrimental to the Favourable Conservation Status of a species. The concept of ‘Favourable Conservation Status’ is central to EU Council Directive 92/43/EEC on the Conservation of natural habitats and of wild fauna and flora (known as the Habitats Directive), whereby the conservation status of a species can be defined as the sum of the influences that may affect the long-term distribution and abundance of its populations. While licenses are issued to exclude bats from roosts under exceptional circumstances, the fate of excluded bats and the impact on their survival and reproduction is not well understood. Studies on big brown bats *Eptesicus fuscus* [10] and little brown bats *Myotis lucifugus* [11] in North America suggest that some species of bats may struggle to find alternative roosts and their reproductive success may be negatively affected by exclusion. However, there is a paucity of data on the impacts of roost exclusion on European bats.

We investigated the effects of exclusion on colonies of *P*. *pygmaeus*, a species that forms large and stable maternity colonies in buildings in Europe [12]. During 2011–13, 87/139 (63%) applications for exclusion licenses in England involved *P*. *pygmaeus* roosts. We used radio-tracking to determine if excluded *P*. *pygmaeus* were able to find suitable alternative roosts and to determine whether their roosting behaviour, home range areas and habitat preferences changed significantly. We examined the potential effects of exclusion on local populations using models that consider population density and a range of potential impacts on reproductive success that might arise from exclusion.

## Materials and Methods

### Site selection

Suitable roost sites in England were identified from exclusion applications submitted to the Bat Conservation Trust (BCT), the NGO which administers applications on behalf of Natural England, the SNCO for England. Sites that had already been granted an exclusion licence were selected whenever possible to avoid excluding bats unnecessarily. Suitable sites were those with large numbers of bats (>100) and where a complete exclusion could be achieved successfully in a day. Exclusion experiments were undertaken in the spring, between 1^st^ May 2012 and 7^th^ June 2013, at five sites across England: Bentham (54°7ʹN, 2°30ʹW), Crakemarsh (52°55ʹN, 1°51ʹW), Shackleford (51°12ʹN, 0°39ʹW), Studland (50°39ʹN, 1°57ʹW) and Willaston (53°17ʹN, 3°0ʹW). With the exception of Studland, all study sites were known maternity roosts, occupied each year by colonies of adult female bats. The roost at Studland contained bats throughout the year, occasionally in high numbers, but had not been confirmed as a maternity roost.

### Roost exclusions

Roost exclusions were performed following method statements issued by Natural England. Temporary one-way exclusion measures were installed at roost exits to allow bats to leave but not re-enter a roost to ensure that none were present immediately prior to the roost being sealed permanently. Examples of the exclusion measures we used are shown in S1 Appendix. The bats at Bentham, Crakemarsh, Shackleford and Willaston were permanently excluded from roosts since licences had been issued for these sites. Bats did not return to three other sites with exclusion licences that we planned to include in the study, so we undertook a temporary exclusion, under licence, at Studland, to enhance sample sizes. Bats were allowed to return to the Studland roost after four days.

### Bat capture and radio-tracking

We used radio-tracking to determine the roosting behaviour, home range areas and habitat preferences of bats for 4 to 7 days pre-exclusion (control) and post-exclusion (exclusion). Bats were caught using hand-held nets as they emerged from roost exits at dusk; their reproductive state was determined at the start of each experiment to ensure that the roost contained neither heavily pregnant or lactating bats with dependent young [13]. Lightweight radio-telemetry tags (PicoPip Ag337, 0.31g: Biotrack Ltd, Wareham, UK) weighing <7% of body mass were fitted to 23 adult female bats at Bentham, 25 at Crakemarsh, 20 at Shackleford, 25 at Studland and 25 at Willaston using an ostomy adhesive solution (Salts Healthcare, Birmingham, UK). All tagged bats were fitted with aluminium bands (3.5 mm: Porzana Ltd, Icklesham, UK) to allow identification of recaptured individuals. Roosting bats were located each day using a R1000 receiver (Communications Specialists Inc., Orange, CA, USA) and a 3-element Yagi antenna to identify alternative roosts. Emergence counts were performed at some alternative roosts using Batbox III D heterodyne bat detectors (Batbox Ltd., Steyning, England) and night vision monoculars (Yukon Advanced Optics Worldwide, Vilnius, Lithuania) to confirm the location of roost cavities and roost exits, and to estimate the number of bats occupying the roost.

Radio-tracking fixes of foraging bats were recorded for up to four hours after sunset when bats were most active. Typically, bats returned to their day roosts within four hours of emergence. We used a standardised ‘shotgun’ approach to collect fix data from foraging bats, whereby four observers at each site recorded locations continuously and sequentially from all bats within detection range using the homing-in method [14–16]. Observers coordinated their movements throughout the areas where bats foraged to maximise overall contact time and ensure that radio fixes were obtained from all or most bats at each site during control and exclusion periods.

All experiments were performed under license from Natural England (licence number: 20120837). The study was approved by the University of Bristol’s Home Office Liaison Team and Ethical Review Group, and was agreed by a Project Advisory Group that included representatives from the BCT, Defra, English Heritage, the National Trust and Natural England. Our data are available in the Dryad Digital Repository, doi:10.5061/dryad.7vp80.

### Home range areas and habitat preferences

The locations of bats were estimated using observer location, bearing and signal strength. Radio fixes were plotted in ArcGIS 10 (Esri Inc., Redland, CA, USA) and imported into Ranges 7 (Anatrack Ltd, Wareham, UK) to calculate colony home ranges (100% minimum convex polygons (MCPs)) and core foraging areas (cluster cores). Cluster polygons were considered the most appropriate minimum-linkage estimators to define core areas since the locations collected from each bat were not independent and so we could not use parametric home range estimators such as ellipses, harmonic means and kernel contours [14–16]. 90% cluster cores were used to define foraging areas since utilisation distribution discontinuities showed that up to 10% of radio fixes were recorded as bats commuted between roosts and foraging areas and so increased estimates of foraging areas disproportionately.

Habitat data were extracted from digital maps developed in-house in ArcGIS 10 using the five broad habitat categories described in Table 1. Habitat preferences were examined for both control and exclusion periods by comparing the habitat composition of areas in which each bat foraged (90% cluster cores) to that available (colony home range; 100% MCP) [15–17]. Used and available habitat compositions were compared using compositional analysis (Compositional Analysis Plus Microsoft Excel tool 6.2, Smith Ecology Ltd, Abergavenny, UK) to determine whether habitats were used in proportion to their availability and to rank habitat types. To meet recommendations that there were ≥ 30 fixes per bat-period i.e. pooled control and pooled exclusion data for each bat [18,19], only data from Bentham (*n* = 4 bats), Crakemarsh (*n* = 14 bats), Shackleford (*n* = 7 bats) and Studland (*n* = 15 bats) were included in analyses of home ranges. To meet the requirement that the number of bats exceeds the number of habitat categories (*n* = 5), only data from Crakemarsh, Shackleford and Studland were used in compositional analyses to determine habitat selection.

**Table 1:** Description of broad habitat types used in analysis of bat habitat preferences.

Habitat	Description
Arable	Ploughed land, cropland and recently reseeded grassland. Includes arable land and grassland in rotation, horticultural land and nurseries, and recently planted and established orchards.
Built-up	Roads, houses and residential land, built-up areas, including areas of commercial retail, industry, high density residential (>40% cover), agricultural buildings, transport areas, restored or active landfill sites, and active or inactive quarries.
Grassland	Any grassland not included under riparian. Includes improved, semi-improved and unimproved grasslands, enclosed meadows and pastures, and amenity grasslands.
Riparian	Open water and marginal vegetation around any water body, including rivers, streams, brooks, lakes, ponds (including operational ponds), reservoirs, aquaculture, estuary and coastal waters, riparian woodland, wet heathland, tall vegetation along water courses, swamp vegetation around pools, and all types of fen and mire.
Woodland	Any woodland not included under riparian. Includes broadleaved, conifer and mixed woodlands, ancient and young stands, forestry scrub, and encompassing all management practices including plantation, restoration, coppice, minimum intervention, etc.

### Data analysis

Sample sizes varied due to loss and failure of tags as the study progressed. We use the term ‘original colony roost’ to define roosts at which we performed exclusions, ‘alternative roost’ to define all roosts other than those at which we performed exclusions, and ‘new colony roost’ to define the alternative roost that most bats moved to following exclusion. We employed an event history-type modelling process to determine if the roosting behaviour of bats was affected significantly by exclusion, whereby we investigated the probability of an event occurring (i.e. the movement of a bat) at each of a series of time-points (i.e. days throughout the experiment). Random effect logistic regression models were fitted to the data to determine (i) whether bats switched roost more frequently following exclusion, and (ii) whether bats used poorer quality roosts more frequently following exclusion. For the first model, the movement of bats over each consecutive day of the experiment was identified by linking the roost location of a bat on one day to its location on the previous day; a bat’s response was either to ‘move’ from or ‘stay’ at a roost. For the second model, to identify roosts that had the potential to serve as a substitute colony roost i.e. capable of supporting a colony of bats equivalent to that excluded, we compared the features of alternative roosts with those of original colony roosts and categorised each alternative roost as either a ‘suitable alternative colony roost’ or an ‘unsuitable alternative colony roost’, and the response of bats was either to ‘move’ from or to ‘stay’ at a roost type. Features that we considered when deciding whether a roost was a ‘suitable alternative colony roost’ included roost type, roost structure, cavity type, location of cavity, and cavity space. Each feature was assessed subjectively because it was often not possible to obtain accurate measurements. Roosts in buildings that were of a similar construction type to the original colony roost, and that offered similar cavity space to that found at the original colony roost, were potential suitable alternative colony roosts. Since *P*. *pygmaeus* prefers to roost close to riparian and woodland foraging habitats [20], and the maximum range span (mean maximum nightly distance from roost to centroid of cluster core foraging area) we recorded prior to exclusion was 3.3 km, roosts further than 3.3 km from foraging areas were considered to be unsuitable alternative colony roosts. The aim of these models was to test whether the response of bats to ‘move’ or to ‘stay’ differed significantly according to period (i.e. control or exclusion; both models) and roost type (i.e. the category of roost in which a bat roosted; model 2). All statistical modelling was performed in MLwiN v2.1 [21].

To determine whether (i) bats were forced to travel further to foraging areas, and (ii) the size of foraging areas changed following exclusion, we calculated mean range spans and size of foraging areas (90% cluster cores) for each bat-period. Data were non-normally distributed and control and exclusion datasets for each response were compared using a Wilcoxon Signed-Rank Test, with significance set at *p* <0.05. To examine if the location of foraging areas changed following exclusion, we calculated the mean percent overlap of control-exclusion pairs of foraging areas for each bat using

$$\frac{\left(\frac{O}{C})+(\frac{O}{E}\right)}{2}$$

where a control foraging area *C* and an exclusion foraging area *E* overlap each other by area *O*. Variability is described throughout as standard deviations (SD) of the mean.

### Population modelling

Currently it is not possible to assess how exclusions might affect the Favourable Conservation Status of *P*. *pygmaeus* because we do not know which are the critical population parameters to monitor. So we developed a stochastic matrix population model that describes *P*. *pygmaeus* demography and provides a method whereby changes in productivity i.e. number of female young reared, and age-specific survival can be simulated and the effects on population growth rate examined. Since there is a thorough review of these techniques [22], the following is a summary of the principles involved in formulating the model. Additional explanation is provided in S2 Appendix.

We constructed a stochastic matrix population model for *P*. *pygmaeus* at the end of the breeding season. We assumed that the sex ratio was equal and only modelled the female part of the population. There were three age classes in the model: the first corresponded to infants produced by the end of a breeding season, the second to individuals in their second calendar year (their first breeding season), and the third to bats in their third calendar year or older (their second plus breeding season). We introduced stochastic variation in age-specific survival to assess the effects of random year-to-year variation in life-cycle parameters. Since there was a lack of information on annual variation in litter size and proportion of individuals breeding each year, we assumed that these variables are constant rather than stochastic. While density dependent factors may be important, they were not considered because any influence on population growth rate was unknown. In the absence of information on movements from outside the local population, the model assumed that populations were closed i.e. there was no immigration or emigration.

The vital rates used for *P*. *pygmaeus* are summarised in Table 2. Additional information on how the vital rates were derived is provided in S2 Appendix. The starting population (colony) was 100 females, chosen to represent a typical colony size, and distributed according to the stable age distribution of the equivalent deterministic model. 1000 realisations were run for an arbitrary time-frame of 500 years. We recorded the mean stochastic growth rate and the proportion of extinct trajectories at the end of the simulation. To investigate the influence of perturbations in vital rates, we altered the annual survival rates (*S*_*1*_, *S*_*2*_ and *S*_*3*_), annual productivity (*P*_*2*_ and *P*_*3*_) and the constituents of productivity (*L*_*2*_, *L*_*3*_, *Alpha*_*2*_ and *Alpha*_*3*_), keeping other rates constant, to examine how changes in each of these rates would influence the population growth rate λ_s_ and to calculate the threshold at which a population of 100 females is likely to become extinct (extinction probability of 1) within 500 years. Matrix calculations were conducted using the program ULM [23].

**Table 2:** Vital rates used in population matrix models for female *Pipistrellus pygmaeus*.

	Vital rate	Estimate (SE)	Source reference
*Annual survival*			
Survival in first year	*S*_*1*_	0.527 (0.095)	[24]*
Survival in second year	*S*_*2*_	0.799 (0.051)	[24]*
Survival in third year plus	*S*_*3*_	0.799 (0.051)	[24]*
*Productivity*			
Mean litter size in second year	*L*_*2*_	1.038	[25–28]
Mean litter size in third year plus	*L*_*3*_	1.038	[25–28]
Proportion breeding in second year	*Alpha*_*2*_	0.930	[29]
Proportion breeding in third year plus	*Alpha*_*3*_	0.930	[29]

* Source data for the common pipistrelle *P*. *pipistrellus*, a closely related cryptic species of the soprano pipistrelle *P*. *pygmaeus*.

## Results

### Roosting behaviour

**Fig 1:**
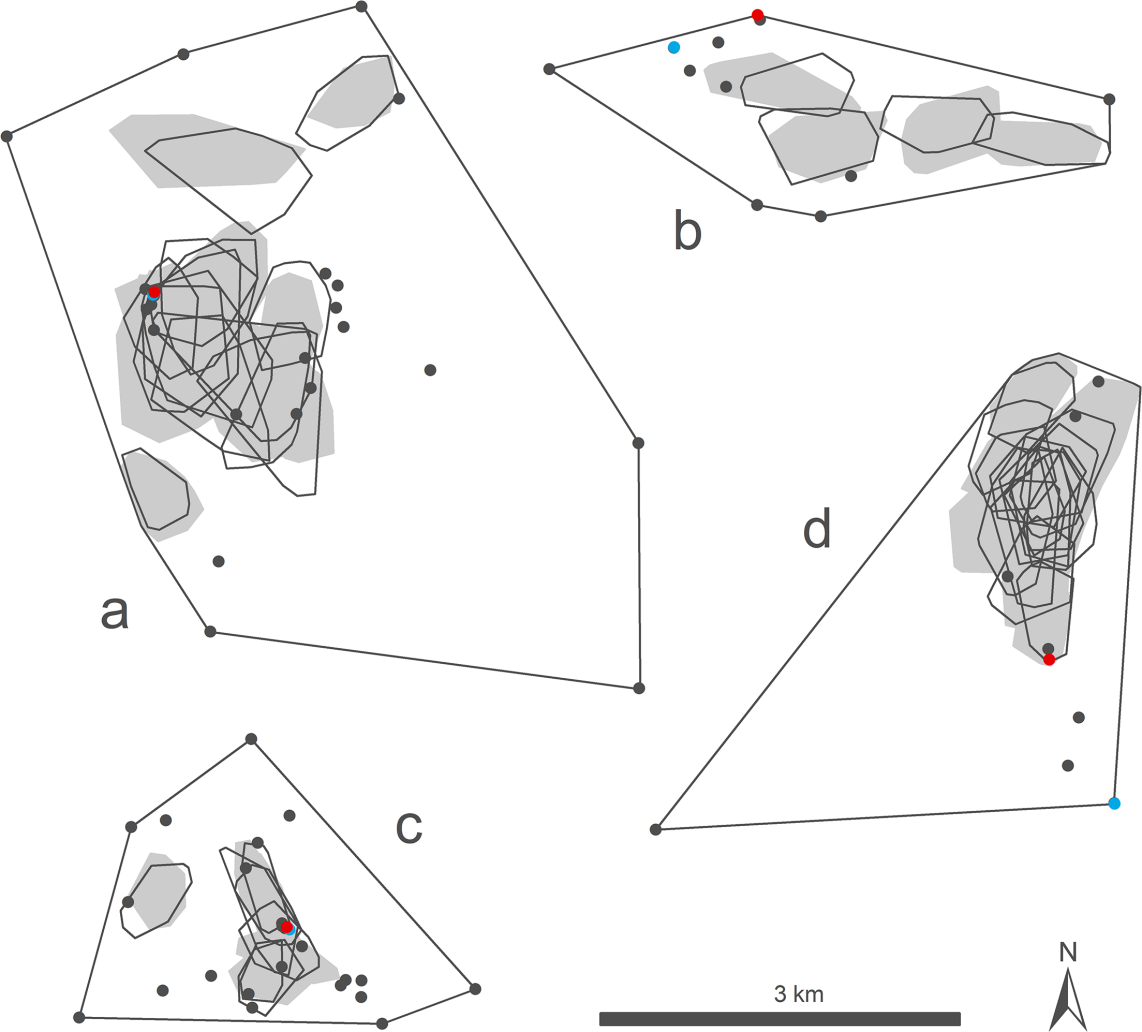
Spatial organisation of *Pipistrellus pygmaeus*. Spatial data collected from female *Pipistrellus pygmaeus* at (a) Crakemarsh (*n* = 25 bats), (b) Bentham (*n* = 23 bats), (c) Shackleford (*n* = 20 bats) and (d) Studland (*n* = 25 bats). Locations of the original colony roost before exclusion (red point), the new colony roost after exclusion (blue point), and alternative roosts (black points) are shown, together with the colony home ranges (100% minimum convex polygon). 90% cluster core foraging areas are shown for bats with ≥30 radio-tracking fixes for both control (solid grey polygons) and exclusion (hollow black polygons) periods (*n* = four bats at Bentham, 14 bats at Crakemarsh, seven bats at Shackleford and 15 bats at Studland).

Across the five study sites, we recorded over 700 day roost fixes from 114 bats and identified 89 alternative roosts (Table 3). Most were in domestic dwellings, ranging from small bungalows to large manor houses. Roosts in uninhabited buildings, such as garages or sheds, industrial warehouses and trees were used to a lesser extent (Table 4). Roosts were typically within a few hundred metres of foraging areas but up to 5 km from the original colony roost (Fig 1). Of the 89 alternative roosts, we considered that 41 (46%) were suitable alternative colony roosts. Of 114 radio-tagged bats, 110 used one or more alternative roosts during the 4 to 7 day control period. Forty-one bats were not recorded in the original colony roost after being caught there i.e. they roosted exclusively in alternative roosts. We performed emergence counts at 24 alternative roosts during control periods; most (*n* = 21) contained relatively few bats (mean 7.6 ± 8.6, range 1–33 bats) compared to the original colony roost from which bats were excluded (190.0 ± 65.2, range 150–300 bats). At Bentham, Shackleford and Studland we identified one alternative roost during the control period that contained a large number (>100) of bats, and tagged bats frequently moved between these roosts and the original colony roosts, indicating that at each of these sites the colony was split between two significant roosts prior to exclusion.

**Table 3:** Radio-tracking data obtained from adult female *Pipistrellus pygmaeus* before (control) and after (exclusion) being excluded from roosts.

Site	Estimated colony size**	Date	*n* bats tagged	*n* alternative roosts used^†^ *		*n* foraging fixes*	
				Control	Exclusion^‡^	Control	Exclusion
Bentham	300	May 2012	23	9	6 (4)	305	143
Crakemarsh	150	May 2013	25	15	19 (10)	507	665
Shackleford	200	May 2013	20	20	11 (6)	340	333
Studland	150	May-June 2013	25	9	3 (3)	709	634
Willaston	150	May 2012	25	17	12 (9)	27	72
Total			118	70	51 (32)	1888	1847

** Estimated maximum number of bats using the original colony roost prior to exclusion.

^†^ *n* roosts used by tagged bats; excludes roost data where bats were not located or where tags had failed.

^‡^ Parentheses = number of roosts that were used during both control and exclusion periods.

* Data accumulated over 4 to 7 day control and exclusion periods.

**Table 4:** Roost use by adult female *Pipistrellus pygmaeus* at Bentham (*n* = 23), Crakemarsh (*n* = 25), Shackleford (*n* = 20), Studland (*n* = 25) and Willaston (*n* = 25). Shows the total number of day roost locations for bats at each site during exclusion experiments, the number of different roost types identified, and the proportional use (parentheses) of each roost type (calculated as the number of incidences that a bat was found roosting in a roost type divided by the total number of diurnal roost locations recorded for the site).

Site	*n* roost fixes	Inhabited building	Uninhabited building	Industrial warehouse	Tree
Bentham	147	8 (0.60)	1 (0.01)	1 (0.38)	1 (0.01)
Crakemarsh	176	10 (0.75)	2 (0.05)	2 (0.01)	10 (0.19)
Shackleford	174	9 (0.59)	5 (0.23)	0 (0.00)	11 (0.18)
Studland	188	3 (0.94)	0 (0.00)	0 (0.00)	6 (0.06)
Willaston	110	16 (0.77)	2 (0.13)	0 (0.00)	2 (0.10)

We successfully excluded all tagged bats from the original colony roost at each site i.e. none returned after the exclusion measures were put in place. Within three days the colony settled on one of the alternative roosts we had identified during the control period and, other than this, we observed no obvious difference in the use of alternative roosts during control and exclusion periods. Following exclusions at Bentham, Shackleford and Studland, the significant alternative roost that we identified during the control period became the new colony roost. At every site, the new colony roost was located within 1.5 km of the original colony roost. At Crakemarsh and Shackleford, the colony moved to a neighbouring property <25 m away. On average, across all sites, bats used a single roost for 2.1 ± 1.3 and 2.0 ± 1.2 consecutive days during control and exclusion periods respectively before changing roost. However, the frequency of roost switching varied considerably between bats, with some switching roost every day and others using a single alternative roost for the duration of the experiment. At Studland, three of the 16 bats that still had functioning radio-tags used the original colony roost on the day after the temporary exclusion measures were removed.

When we fitted multilevel logistic regression models to transition data, we found no effect of exclusion on frequency of roost movements (Wald χ^2^ (1) = 0.249, *p* = 0.62) i.e. bats changed roost equally often during control and exclusion periods. When we considered roost type, we found that bats were significantly less likely to move from a ‘suitable’ colony roost than from an ‘unsuitable’ colony roost (Wald χ^2^ (1) = 9.566, *p* = 0.002) i.e. bats stayed longer in colony-type roosts before moving. Following exclusion there was a small but significant increase in the likelihood that bats would roost in a ‘suitable’ colony roost (Wald χ^2^ (1) = 12.212, *p* <0.001); this increased from 79% probability during control periods to 87% probability after exclusion.

### Foraging behaviour

Across the five study sites we recorded over 3700 foraging fixes from 103 bats (Table 3). Range data for control and exclusion periods (Table 5) show that, on average, bats foraged close to day roosts and used only a small portion (4.2 ± 1.8%, *n* = 40 bats) of the colony home range area for foraging. At Crakemarsh, Shackleford and Studland, the foraging areas of individual bats were highly clustered and overlapping, suggesting a sharing of resources at these sites (Fig 1). At Bentham, foraging areas were clustered but non-overlapping; the lack of overlap may be due to the small sample size (*n* = 4 bats). Bats foraged in similar-sized core areas (control mean = 43.6 ± 20.5 ha; exclusion mean = 46.5 ± 21.8 ha; *Z* = -1.358, *p* = 0.175) that were located in the same place (mean overlap of control and exclusion core foraging areas = 76.4 ± 9.7%; minimum 51.2, maximum 88.4) and bats travelled similar distances to reach foraging areas (control mean = 1.5 ± 0.9 km; exclusion mean = 1.48 ± 1.0 km; *Z* = -0.704, *p* = 0.482).

**Table 5:** Colony home range areas (100% MCPs), foraging areas (90% cluster cores) and range spans (mean maximum nightly distance from roost to centroid of 90% cluster core) for 40 adult female *Pipistrellus pygmaeus* radio-tracked before (control) and after (exclusion) being excluded from roosts.

Site	Date	*n* bats	Period	Colony home range (ha)*	Foraging area (ha)*	Range span (km)*
Bentham	May 2012	4	Control	482.0	40.3 ± 5.4	1.72 ± 0.98
			Exclusion	491.2	38.7 ± 5.8	1.75 ± 1.37
Crakemarsh	May 2013	14	Control	1856.8	61.6 ± 22.2	0.74 ± 0.25
			Exclusion	2071.1	66.4 ± 22.6	0.81 ± 0.33
Shackleford	May 2013	7	Control	493.3	23.2 ± 4.6	0.70 ± 0.51
			Exclusion	493.3	23.0 ± 6.2	0.46 ± 0.05
Studland	May-June 2013	15	Control	935.5	37.2 ± 11.1	2.45 ± 0.49
			Exclusion	643.9	40.9 ± 10.0	2.53 ± 0.41

* Mean ± SD, calculated as mean (*n* bats) of means (*n* bat-days).

Compositional analyses to determine habitat preferences of bats at each site revealed that they consistently preferred to forage in riparian habitat, followed by woodland, over other habitat types (Table 6). Arable habitat and built-up areas consisting mainly of medium density residential housing (>40% cover) were preferred least. Habitat preferences of bats were the same during control and exclusion periods (Table 6).

**Table 6:** Habitat preferences of adult female *Pipistrellus pygmaeus* (Crakemarsh *n* = 14 bats; Shackleford *n* = 7 bats; Studland *n* = 15 bats) during control and exclusion periods. Habitat categories to the left of > are selected over those to the right, with >>> showing a significant difference between adjacent habitat types.

Site	Period	Ranked habitat types									*p* *
Crakemarsh	Control	Riparian	>	Woodland	>	Grassland	>	Built-up	>	Arable	<0.001
	Exclusion	Riparian	>	Woodland	>	Grassland	>	Built-up	>	Arable	<0.01
Shackleford	Control	Riparian	>	Woodland	>>>	Grassland	>	Arable	>	Built-up	<0.05
	Exclusion	Riparian	>	Woodland	>>>	Grassland	>	Built-up	>	Arable	<0.01
Studland	Control	Riparian	>>>	Woodland	>>>	Grassland	>	Built-up	>	Arable	<0.001
	Exclusion	Riparian	>>>	Woodland	>>>	Grassland	>	Built-up	>	Arable	<0.001

* *p*-values <0.05 show selection of habitat types is non-random.

### Population model

**Fig 2:**
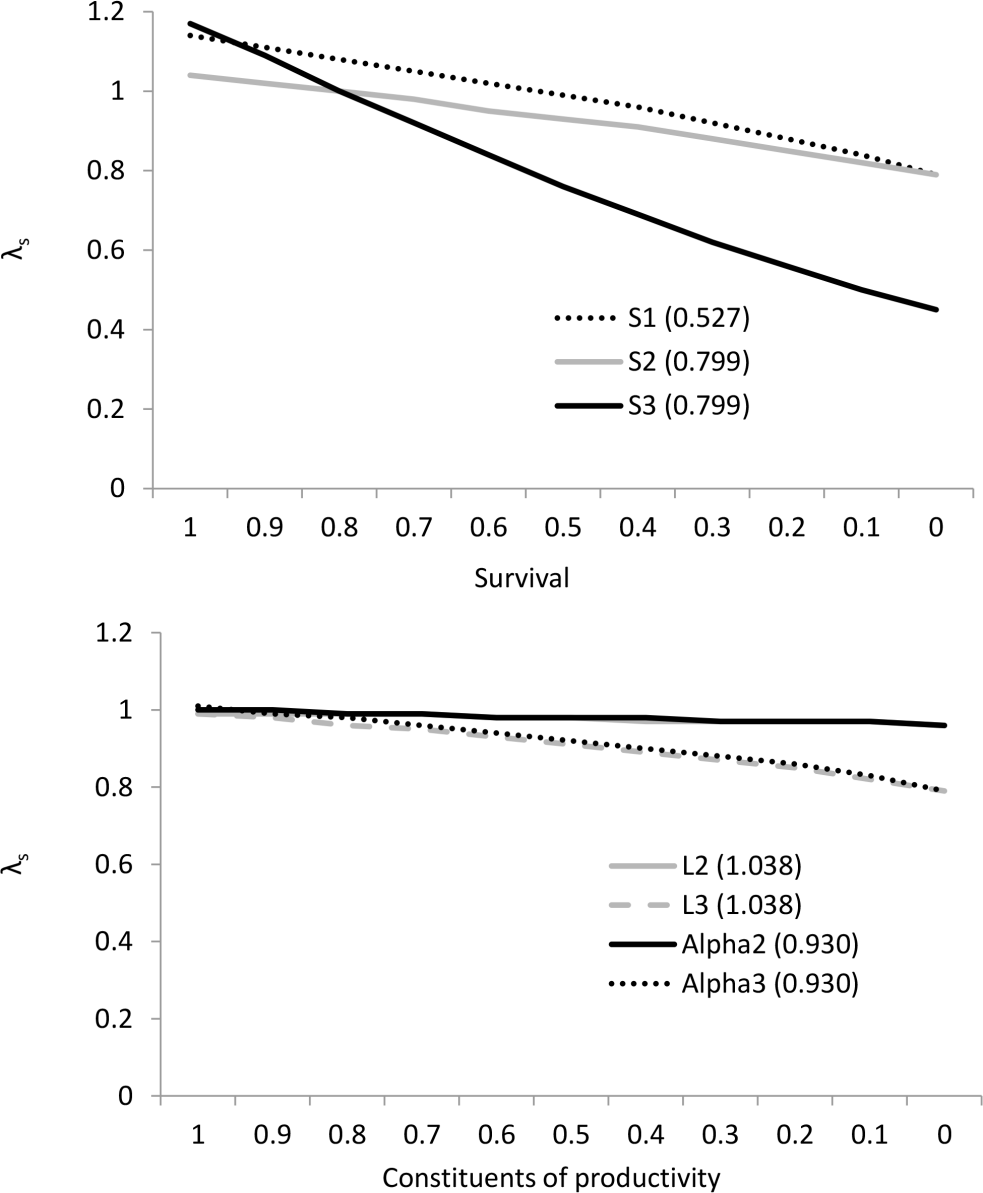
Modelled population growth rates of *Pipistrellus pygmaeus*. Effects of changing age-specific annual survival rates (top) and the constituents of productivity (bottom) on population growth rate of female soprano pipistrelles; the vital rates used are shown in brackets. In the absence of perturbation, the mean stochastic growth rate λ_s_ was 0.997 i.e. essentially stable.

The projection matrix model derived from the vital rates in Table 2 gave a mean stochastic population growth rate λ_s_ of 0.997 i.e. essentially stable, where none of the 1000 trajectories was extinct after 100 years. First year survival *S*_*1*_, second year survival *S*_*2*_ and survival from three years onwards *S*_*3*_ were the most important parameters contributing to population growth (Table 7, Fig 2). The elasticity of *S*_*1*_ and *S*_*2*_ are approximately equal to the sum of the combined elasticities of productivity for all age groups. Individual components of productivity i.e. mean litter size of bats breeding in their second year (*L*_*2*_), and third year plus (*L*_*3*_), and the proportion of individuals breeding in their second year (*Alpha*_*2*_) and third year plus (*Alpha*_*3*_) have comparatively small elasticities. Therefore, changes in these parameters are likely to have a comparatively small effect on population growth rate (Table 8, Fig 2). The critical threshold of the population parameters below which a population of 100 females is likely to become extinct within an arbitrary 500 years is shown in Table 9.

**Table 7:** Elasticities and sensitivities of matrix cells derived from the population projection matrices for female *Pipistrellus pygmaeus*.

	Elasticity	Sensitivity
Annual survival		
*S*_*1*_	0.17	0.32
*S*_*2*_	0.17	0.21
*S*_*3*_	0.66	0.83
Productivity		
*P*_*2*_	0.04	0.08
*P*_*3*_	0.14	0.28

**Table 8:** Elasticities and sensitivities for the constituents of productivity derived from the population projection matrices for female *Pipistrellus pygmaeus*.

	Vital rate	Elasticity	Sensitivity
Mean litter size in second year	*L*_*2*_	0.04	0.04
Mean litter size in third year plus	*L*_*3*_	0.13	0.13
Proportion breeding in second year	*Alpha*_*2*_	0.04	0.04
Proportion breeding in third year plus	*Alpha*_*3*_	0.14	0.14

**Table 9:** Critical threshold of population parameters for female *Pipistrellus pygmaeus*, below which a population of 100 females is likely to become extinct within an arbitrary 500 years; figures in brackets show the vital rates used in the population models.

	Vital rate	Critical values (vital rates)
*Annual survival*		
Survival in first year	*S*_*1*_	0.46 (0.527)
Survival in second year	*S*_*2*_	0.70 (0.799)
Survival in third year plus	*S*_*3*_	0.77 (0.799)
*Productivity*		
Mean litter size in second year	*L*_*2*_	0.20 (1.038)
Mean litter size in third year plus	*L*_*3*_	0.85 (1.038)
Proportion breeding in second year	*Alpha*_*2*_	0.18 (0.930)
Proportion breeding in third year plus	*Alpha*_*3*_	0.77 (0.930)

With a starting population of 100 females, with all other parameters remaining constant, annual survival would need to decline by 7% (53% to 46%) for individuals less than a year old (*S*_*1*_) to bring about population extinction (extinction probability = 1) over an arbitrary 500 year period, by 10% (80% to 70%) for individuals in their second year (*S*_*2*_), or by 3% (80% to 77%) for individuals in their third year or older (*S*_*3*_). In terms of the constituents of productivity, mean litter size of individuals breeding in their second year (*L*_*2*_) and in their third year plus (*L*_*3*_) would need to decline by 0.84 (1.04 to 0.20 young) and by 0.19 (1.04 to 0.85 young), respectively. The proportion of individuals breeding in their second year (*Alpha*_*2*_) and in their third year plus (*Alpha*_*3*_) would need to decline by 75% (93% to 18%) and 16% (93% to 77%), respectively (Table 9).

While the number of years of simulation here is arbitrary, the results highlight that demographic monitoring should focus on obtaining robust estimates for adult survival, with a lower priority on obtaining robust estimates of first and second year survival, mean litter size of bats in their third year plus, and the proportion of individuals breeding in their third year plus.

## Discussion

Our data show that in spring and early summer *P*. *pygmaeus* forms fission-fusion societies, with bats moving with varying frequency between one or two main roosts, which sustain relatively high numbers of bats, and a large number of alternative roosts. *P*. *pygmaeus*, along with probably all temperate-zone bat species, enters regulated torpor to maximise energy conservation when confronted with periodic food shortages and/or adverse environmental conditions [30–32]. The use of a wide variety of alternative roosts in spring, ranging from individual roosts behind ivy on trees to substantial colony roosts in inhabited i.e. heated dwellings, may reflect the variety of roost microclimates needed by soprano pipistrelles to facilitate this behaviour. Factors such as predation risk, parasite load within roosts, social behaviour and anthropogenic disturbance may also influence roost switching [33–36].

Nearly half (46%) of the alternative roosts used by radio-tagged bats were considered suitable for supporting colonies equivalent in size to those excluded. At all sites there was a cluster of these colony-type roosts close to the original colony roost, often in neighbouring buildings of similar construction. Some of these roosts may serve as the main colony roost at different times of the year, or in different years. The bats did not return to three sites we had earmarked for experiments, probably because they used an alternative colony roost nearby.

We were successful at excluding bats from roosts. The method statements currently issued with licences in the United Kingdom provide advice that is appropriate for performing exclusions safely and effectively. We detected no change in the use of alternative roosts by bats following exclusion and all tagged bats that we excluded found alternative roosts nearby. At all sites, colonies congregated in an alternative roost within three days and these roosts were within 1.5 km of the original roost i.e. bats did not emigrate following exclusion. We observed no change in the frequency of roost movements and bats were not forced to use roosts that we perceived to be unsuitable colony roosts more often. Bats also continued to forage in the same areas. Our data on roosting and foraging behaviour are similar to those for *P*. *pygmaeus* elsewhere [15,17,20,37,38], and so we predict that other *P*. *pygmaeus* colonies are likely to respond similarly to exclusion.

Although we observed no significant change in the behaviour of bats during our short-term experiments, there is little information on the longer-term impacts of exclusion on productivity and survival rates for *P*. *pygmaeus*, or indeed any bat species. To our knowledge, the only study to examine the demographic consequences of roost exclusion is for the big brown bat, *Eptesicus fuscus*. Despite individuals relocating to roosts nearby, mean litter size was significantly lower (56% reduction) following exclusion (0.86 ± 0.30 at control sites; 0.38 ± 0.30 following exclusion) [10]. A change of similar magnitude in *L*_*2*_ could have profound consequences for *P*. *pygmaeus* populations.

If the roosting behaviour that we observed during our experiments is indeed shared among soprano pipistrelles generally, and colonies have a large number of alternative roosts that they could move to quickly when excluded from one roost, this may be adequate to buffer populations against the limited number of exclusions currently licensed in the United Kingdom. However, we do not know what the impact on local populations would be from multiple exclusion events. Each year, exclusions are conducted at a large number of bat roosts in the United Kingdom during development work but only limited information is provided on the consequences of mitigation [39]. On a larger scale, the impacts of exclusions are likely to be more substantial. While we believe that exclusion is perhaps most likely to have an impact on demographic rates through a reduction in productivity, we have no information on the impact of exclusion on survival. However, our modelling demonstrates that fairly small reductions in annual survival, particularly adult survival, would result in a declining population growth rate, at least for the year following exclusion.

The timing of exclusions is critical to avoid causing disturbance to heavily pregnant bats, dependant young and hibernating bats, and the current recommendation in England is that exclusions are performed during October or April to avoid disturbing bats at sensitive times of the year. Our experiments were conducted closer to the summer breeding period than is normally permitted, but we observed no significant detrimental impact on the bats. Extending licensing windows to permit exclusions after April may be desirable, providing allowances are made for annual variations in weather that affect the timing of pregnancy and adequate support is available to ensure pregnancy is not too far advanced. Although we monitored the bats over relatively short time periods, soprano pipistrelles radio-tracked in southern England [20] also used several different roosts during spring and early summer, and continued to move between roosts over extended tracking periods, so we infer that the response of bats to exclusion will be similar throughout this period. It is likely that roost-switching will become less frequent during lactation given the difficulties and costs incurred by mothers transporting pups to new sites.

Importantly, our findings should be treated as species-specific and should not be extrapolated to other species, including *P*. *pipistrellus* which, despite being a cryptic species of *P*. *pygmaeus*, shows distinct behavioural differences [15,17,37,38,40]. The colonies that we studied were typical in size (150–300 bats) for *P*. *pygmaeus* [20,40]. While colonies of more than 1500 bats are known, we predict that bats will respond similarly to exclusions irrespective of colony size. Further research is, however, needed to examine the impact of exclusion on larger *P*. *pygmaeus* colonies. Although our conclusions are based on short-term responses by the bats, arguably these are useful proxies for detriment. However, long-term studies comparing productivity and survival in excluded versus control (non-excluded) populations are needed to draw definitive conclusions about the long-term consequences of exclusion on Favourable Conservation Status. Further research is also needed to determine the impact of exclusions on other British species that are frequently affected by exclusions, for example *P*. *pipistrellus* and the brown long-eared bat *Plecotus auritus*.

## Conclusions

We have shown that, following exclusion, a soprano pipistrelle colony is able to relocate to a new colony roost quickly and without any obvious short-term impact on behaviour or welfare. The availability of suitable alternative roosts is critical in determining the impact of future exclusions on these bats, although soprano pipistrelles are able to make use of a wide variety of both natural and man-made structures for roosting. We cannot be certain what effect exclusion has on the Favourable Conservation Status of *P*. *pygmaeus* because we have no measure of the long-term impact on survival and productivity. While we predict that any impact is likely to be small because few exclusions are licensed at present, the impacts of higher rates of roost destruction may be of concern and require further investigation.

## Supporting Information

S1 Appendix Examples of Exclusion Procedures. (DOCX)

S2 Appendix Additional Explanation of Population Modelling Procedures. (DOCX)
