# Supplementary material for: Managing Conflict between Bats and Humans: The Response of Soprano Pipistrelles (Pipistrellus pygmaeus) to Exclusion from Roosts in Houses
Source: PLoS One. 2015 Aug 5;10(8):e0131825. doi: 10.1371/journal.pone.0131825 (PMC4526527; doi:10.1371/journal.pone.0131825)
Supplement: S1 Appendix — (DOCX) [file pone.0131825.s001.docx]

**S1 Appendix: Examples of Exclusion Procedures**

Below are three examples of different techniques used during the experiments to exclude soprano pipistrelle bats from roosts. In all three cases, all the bats were excluded successfully from the roost in a single day. Successful exclusions rely on one-way measures at roost access points to allow bats to leave but not return. These temporary installations are eventually removed and roost exits are permanently sealed.

| **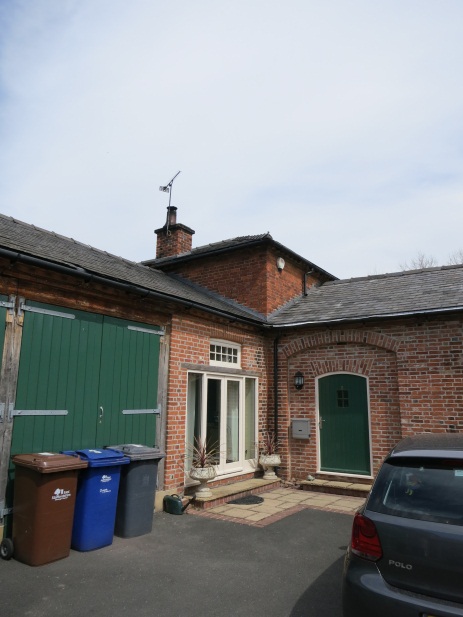** | 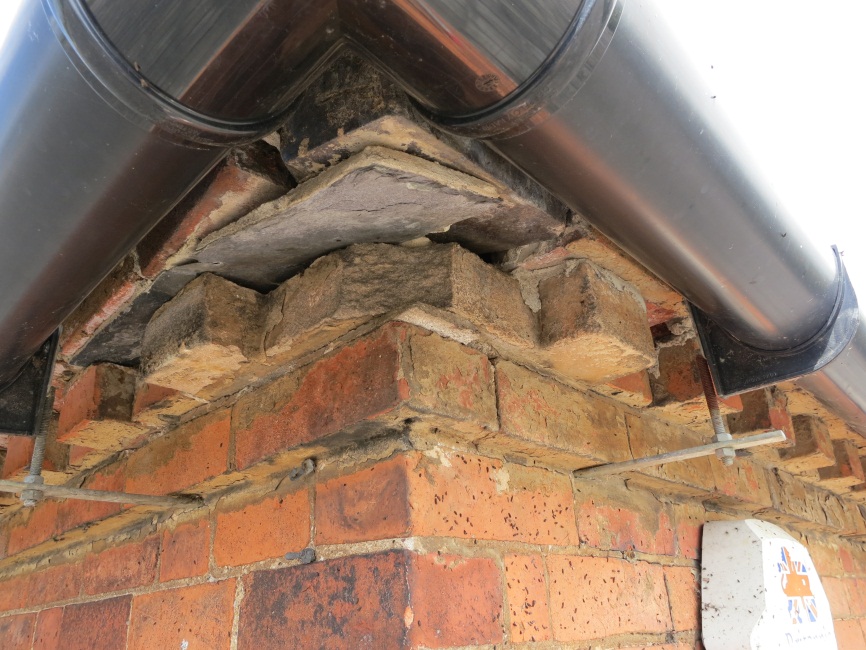 |
| --- | --- |
| **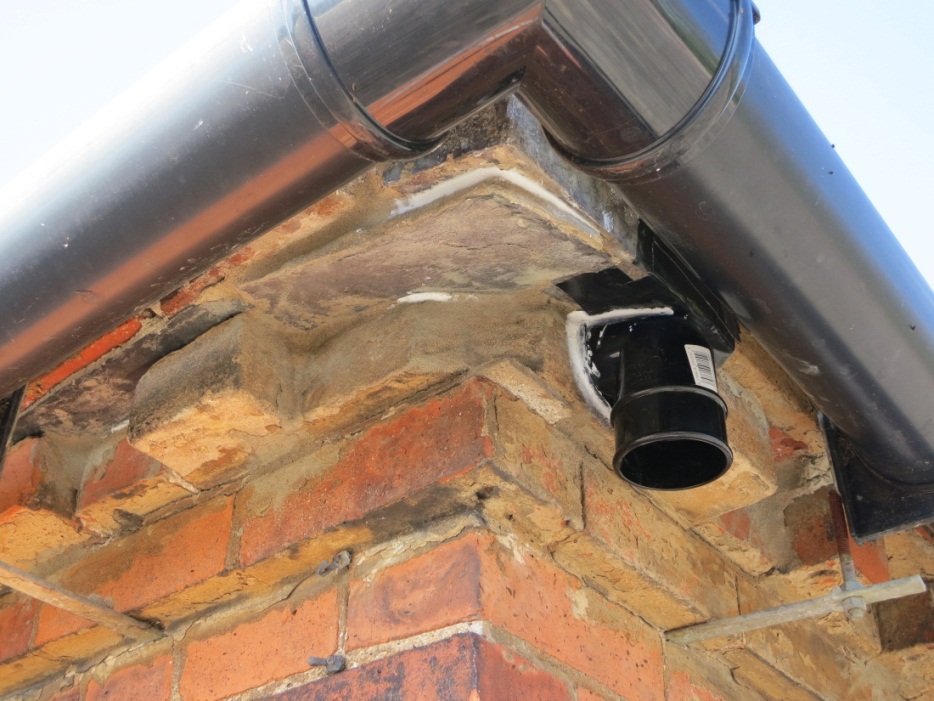** | **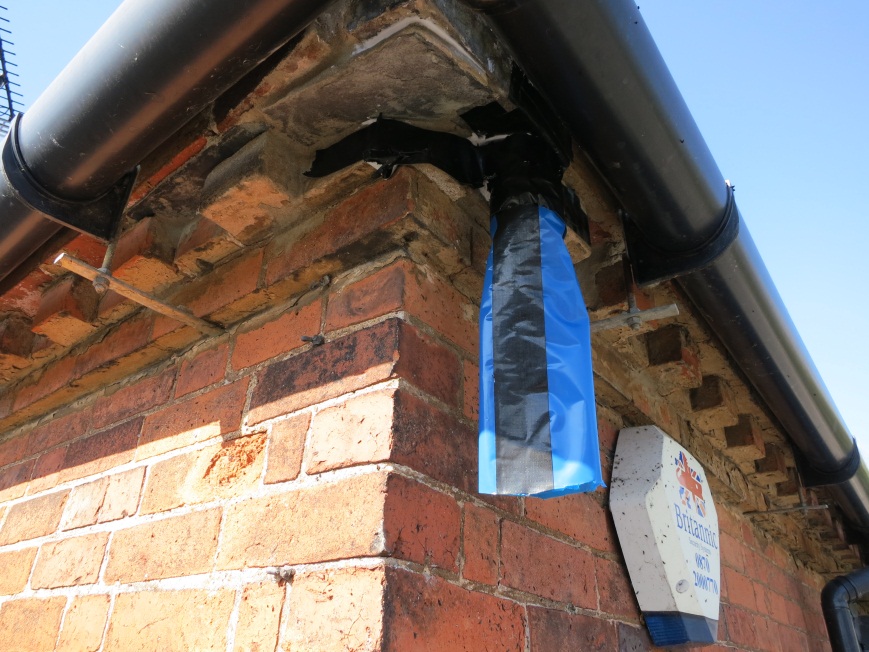** |

One-way exclusion measure installed at Crakemarsh. In this example, gaps in the brickwork were filled in and one hole was left open to create a single exit point for the bats. Plastic guttering and a plastic bag ‘shoot’ create an effective one-way exclusion measure for bats. Images: Matt Zeale

| **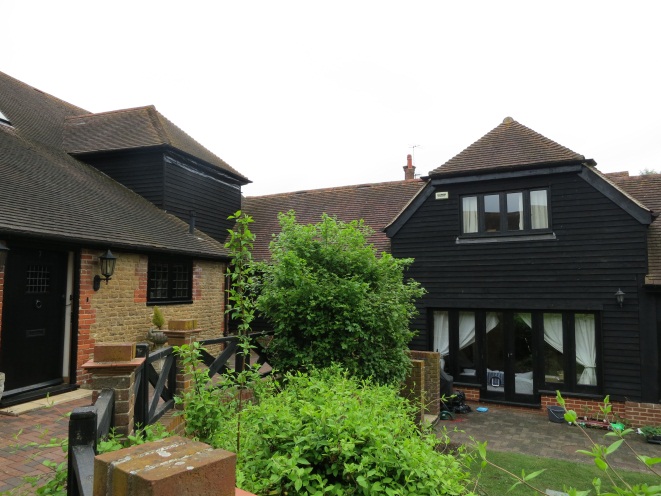** | **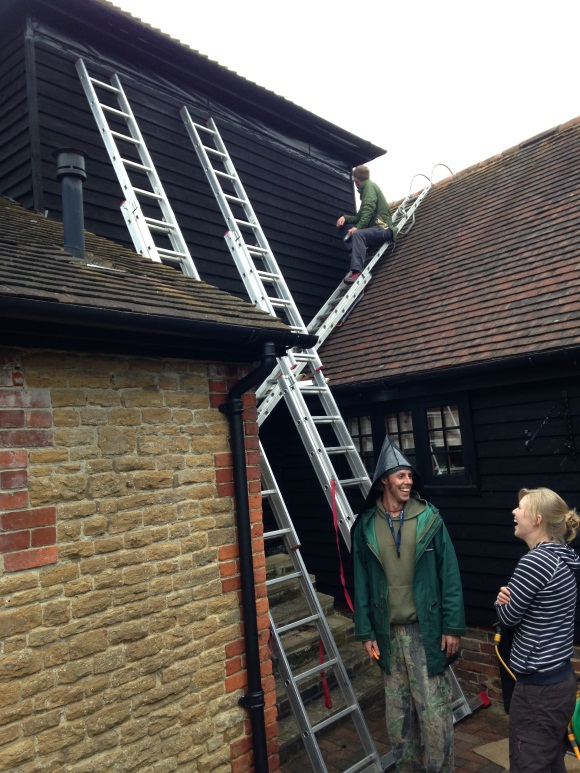** |
| --- | --- |
| **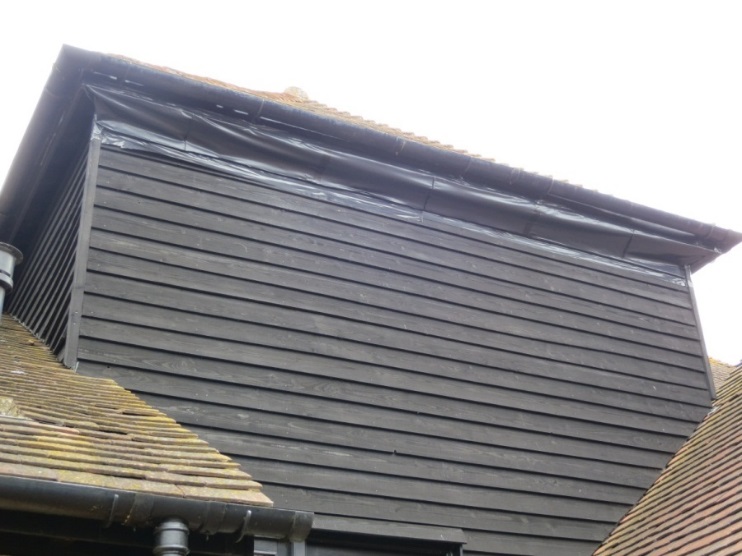** |  |

One-way exclusion measure installed at Shackleford. Plastic ‘flashing’ can be used to great effect to exclude bats from roosts where there are multiple roost exits under the eaves along the length of a building. Images: Matt Zeale

| 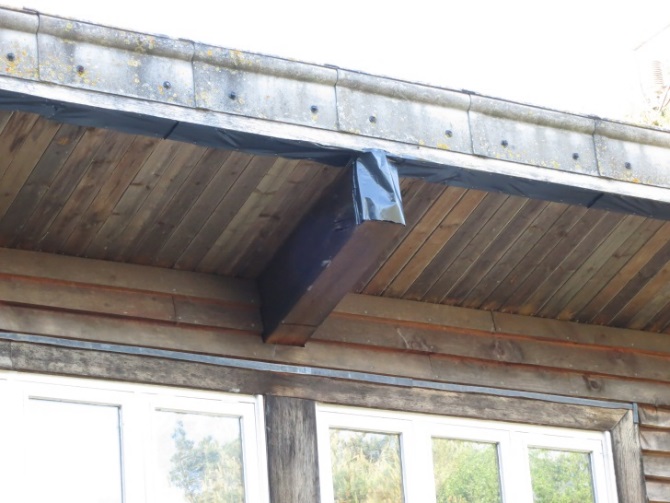 | 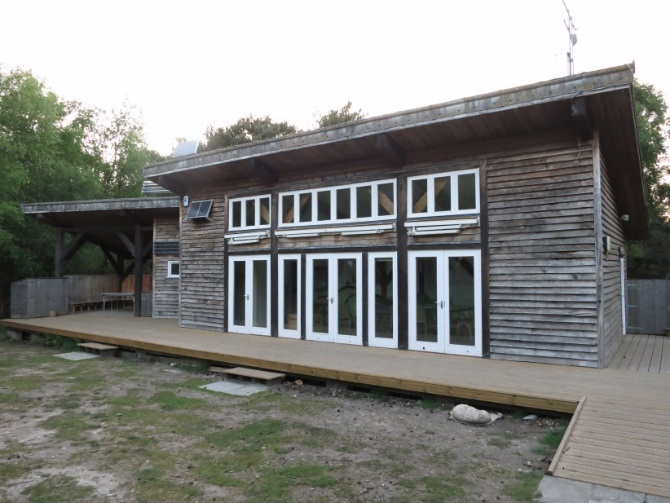 |
| --- | --- |

One-way exclusion measure installed at Studland. On buildings that have a large number of potential roost access points, plastic sheeting is a cheap material that can be used to seal off large parts of the building while leaving simple one-way measures at the key exit points identified during emergence surveys. Images: Matt Zeale
