# Supplementary material for: Managing Conflict between Bats and Humans: The Response of Soprano Pipistrelles (Pipistrellus pygmaeus) to Exclusion from Roosts in Houses
Source: PLoS One. 2015 Aug 5;10(8):e0131825. doi: 10.1371/journal.pone.0131825 (PMC4526527; doi:10.1371/journal.pone.0131825)
Supplement: S2 Appendix — (DOCX) [file pone.0131825.s002.docx]

**S2 Appendix: Additional Explanation of Population Modelling Procedures**

For our analyses, population size *n* was defined as the number of females in a population and was the sum of the numbers in each age class

*n = n_1_ + n_2_ + n_3_ …. n_i_* (1)

where n*_i_* is the number of females in age class *i*. A population with three age classes can be described by a set of difference equations, where *P_i_* are age-specific productivities i.e. the number of female young produced at each age class *i* and *Si* are age-specific survival probabilities of age class *i*, such that

*n*_1_(*t*+1) = *P*_2_*n*_2_(*t*) + *P*_3_*n*_3_(*t*)

*n*_2_(*t*+1) = *S*_1_*n*_1_(*t*) (2)

*n*_3_(*t*+1) = *S*_2_*n*_2_(*t*) + *S*_3_*n*_3_(*t*)

So a generic age structured bat population can be represented as a life-cycle graph thus

The difference equations describing the bat population can be written more simply in matrix form

**n**(*t*+1) = **An**(*t*), (3)

where **n** is an age distribution vector

**n**(*t*) =$\left[\begin{matrix} \text{ }n_{1}\text{(t) } \\ \text{ n}_{2}\text{(t)} \\ \text{ n}_{3}\text{(t)} \end{matrix}\right]$, (4)

and **A** is a population projection matrix as described by [1,2]. The elements of the matrix can be obtained from the difference equations or from the life-cycle graph, so that

= **$\left[\begin{matrix} P_{1} & P_{2} & P_{3} \\ S_{1} & 0 & 0 \\ 0 & S_{2} & S_{3} \end{matrix}\right]$** (5)

This is known as an extended Leslie matrix; it assumes the vital rates in the matrix are equal after age class two.

By repeatedly multiplying the matrix **A** by the vector **n** a series of vectors are obtained that stabilise out after a few generations, so they differ from each other by a scalar factor. This factor λ, is the dominant eigenvector of the population projection matrix **A**, and is equal to the population growth rate, so that

**n**(*t*+1) = λ**n**(*t*), (6)

where

λ = *e^r^* , (7)

*r* being the per capita rate or intrinsic rate of population growth. Successively multiplying **n** by **A** also results in the vector **w** that is proportional to the stable age structure of the population.

**w** =$\left[\begin{matrix} \text{ }w_{1}\text{ } \\ \text{ }w_{2} \\ \text{ }w_{3} \end{matrix}\right]$, (8)

The stable age structure **w** and λ can be used to determine the population size **n** at any time *t*, thus

**n**(*t*) = λ*^t^***w**. (9)

Substituting this into (3) gives

λ^t+1^**w** = λ*^t^***Aw**. (10)

A scalar λ and vector **w** that satisfy this equation are the dominant eigenvalue and right eigenvector of the matrix **A**, respectively. These must satisfy

(**A** - λ**I**) **w** = 0, (11)

where **I** is the identity matrix. **w** has a nonzero solution only if the determinant of the matrix.

**A** - λ**I** equals zero, hence the value of λ and **w** can be solved using the characteristic equation

det (**A** - λ**I**) = 0, (12)

The characteristic equation can be solved using polynomial expansion, which for bats here with a matrix of three by three, produces seven eigenvalues and their associated eigenvectors, where λ is the dominant eigenvalue. This also produces a solution for the left eigenvector **v** of the matrix **A** which is known as the reproductive stage vector and represents the contribution of each age class to the population, so that

**v**^*^**A** = λ**v**^*^, (13)

where **v**^*^ is the transpose of **v**. The left and right eigenvectors can be scaled so that their scalar product equals one, so for the eigenvalue λ

〈**w,v**〉 = **v**^*^**w** = 1, (14)

where 〈**w,v**〉 is the scalar product of **w** and **v**. This allows calculation of the effect that a small additive change in any of the vital rates of the population projection matrix **A** would have on λ, so that

$s_{ij}=\frac{\partial\lambda}{\partial\text{a}_{\text{ij}}}=v_{i}w_{j},$ (15)

where s_ij_ is the sensitivity of λ to changes in the matrix element a*_ij_*. The sensitivity of λ to changes in other parameters can then be calculated for any other vital rate (*x*).

$\frac{\partial\lambda}{\partial\text{ x}}=\sum_{i,j} \frac{\partial\lambda}{\partial\text{a}_{\text{ij}}}\frac{\partial\text{a}_{\text{ij}}}{\partial\text{x}}.$ (16)

Often more important in conservation and management is to determine the proportional effect that a small change in each vital rate would have on λ. This can be examined using elasticity analysis, where

$e_{ij}=\frac{a_{ij}}{\lambda}\frac{\partial\lambda}{\partial\text{a}_{\text{ij}}}.$ (17)

where the elasticity to other parameters can be calculated by

$e(x)=\frac{x}{\lambda}\frac{\partial\lambda}{\partial x}$ (18)

$\text{ }=\frac{x}{\lambda}\sum_{i,j} \frac{\partial\lambda}{\partial a_{ij}}\frac{\partial a_{ij}}{\partial x}.$ (19)

**Estimating vital rates for soprano pipistrelles**

Several studies provide some information on litter size for *P. pygmaeus* in the United Kingdom [3-6]. Collectively, they recorded three sets of twins from 78 females i.e. a 3.8% rate of twinning, which we used here. We assumed an equal sex ratio at birth and that female bats can start breeding at one year of age, such that

$P_{i}=L_{i}*alpha_{i}$ (20)

where *P_i_* is the productivity of age class *i* (where *i* = {1, 2}), *L_i_* is the mean litter size and *alpha_i_* is the proportion of age class *i* breeding*.* The productivity of bats in their first year (i.e. infants) will be 0. In one study 110 of 118 (93%) female *P. pygmaeus* examined at several points overwinter had been inseminated [7]. While we did not know the proportion of second year versus older individuals breeding in a given year, we assumed this was 93% for both age-classes, and productivity was expressed as

$\begin{matrix} P_{1}=0 \\ \\ P_{2}=L_{2}*alpha_{2} \\ \\ P_{3}=L_{3}*alpha_{3} \end{matrix}$ (21)

There were no robust survival estimates for *P. pygmaeus*; the data available [e.g. 8] are now believed to be underestimates of true survival due to methodological drawbacks of the approaches used at the time. Limitations of these data include unreliable assumptions about age-distribution and population stability, and a failure to account for variable recapture probabilities [9]. So we used survival estimates for the closely related *P. pipistrellus* [9]; standard errors of these data suggest that annual survival varied by about 5-10%. Looking at studies on other species of bats, first year survival varied by about 5% between year for Leisler’s bat *Nyctalus leisleri* in Switzerland, and adult survival by 12% [10]. Adult survival varied by about 10% between years for the long-fingered bat *Myotis capaccinii* in Greece [11], while adult survival varied by about 16% in the Isabelline serotine *Eptesicus isabellinus* in Spain [12]. Since the annual variation in survival was broadly similar across studies, we assumed a mean of 11%.

**References**

1. Leslie PH (1945) On the use of matrices in certain population mathematics. Biometrika 33: 183-212.

2. Leslie PH (1948) Some further notes on the use of matrices in population mathematics. Biometrika 35: 213-245.

3. Hughes PM, Speakman JR, Jones G, Racey PA (1989) Suckling behaviour in the pipistrelle bat (*Pipistrellus* *pipistrellus*). J Zool 219: 665-670. doi: 10.1111/j.1469-7998.1989.tb02607.x.

4. Bishop CM, Jones G, Lazarus CM, Racey PA (1992) Discriminate suckling in pipistrelle bats is supported by DNA fingerprinting. Mol Ecol 1: 255-258. doi: 10.1111/j.1365-294X.1992.tb00185.x.

5. de Fanis E (1994) Cues used in communication by Microchiropteran bats. University of Bristol: PhD thesis.

6. de Fanis E, Jones G (1996) Allomaternal care and recognition between mothers and young in pipistrelle bats (*Pipistrellus pipistrellus*). J Zool 240: 781-787. doi: 10.1111/j.1469-7998.1996.tb05324.x.

7. Racey PA (1974) Ageing and assessment of reproductive status of pipistrelle bats, *Pipistrellus pipistrellus*. J Zool 173: 264-271. doi: 10.1111/j.1469-7998.1974.tb03136.x.

8. Gerell R, Lundberg K (1990) Sexual differences in survival rates of adult pipistrelle bats (*Pipistrellus pipistrellus*) in south Sweden. Oecologia 83: 401-404.

9. Sendor T, Simon M (2003) Population dynamics of the pipistrelle bat: effects of sex, age and winter weather on seasonal survival. J Anim Ecol 72: 308-320. doi: 10.1046/j.1365-2656.2003.00702.x.

10. Schorcht W, Bontadina F, Schaub M (2009) Variation of adult survival drives population dynamics in a migrating forest bat. J Anim Ecol 78: 1182-1190. doi: 10.1111/j.1365-2656.2009.01577.x.

11. Papadatou E, Butlin RK, Pradel R, Altringham JD (2009) Sex-specific roost movements and population dynamics of the vulnerable long-fingered bat, *Myotis capaccinii*. Biol Conserv 142: 280-289. [doi: 10.1016/j.biocon.2008.10.023](http://dx.doi.org/10.1016/j.biocon.2008.10.023).

12. Papadatou E, Ibáñez C, Pradel R, Juste J, Gimenez O (2011) Assessing survival in a multi-population system: a case study on bat populations. Oecologia 165: 925-933. doi: 10.1007/s00442-010-1771-5.
